# A Lecture Delivered in King's College, London, on Wednesday, 14 March, 1832

**Published:** 1832-05

**Authors:** Gilbert T. Burnett

**Affiliations:** Professor of Botany in the College and to the Medico-Botanical Society, &c. &c. &c.


### BOTANY.

*A Lecture delivered in King's College, London, on Wednesday, 14th March, 1832, by* Gilbert T. Burnett, F.L.S., M.B. R.I. R.C.S., Professor of Botany in the College and to the Medico-Botanical Society, &c. &c. &c.

Gentlemen: Whether Botany as the science which treats of plants, or Plants as the subject-matter of the science, be made our first especial theme, is a point of little comparative importance; for the things studied and the study are, of necessity, so conjoined and blended together, that they always must be connectedly pursued, even though each in turn, as priority is yielded, may seem to be used chiefly in illustration of the other. Knowledge, and the things known, are abstractedly distinct, and hence should ever in theory be distinguished; but in practice they can never be divided.

Did time permit, it might be well to discuss both schemes at once; and to offer an outline of both systems in a single lecture; for the more aspects under which any object can be viewed, the more familiar do we become with its several parts, and the more intimately acquainted with its bearings: but as an hour is scarcely sufficient for even the most cursory glance at one, they must be consecutively pursued, and as the last Course was opened with a practical demonstration of plants as they are found in nature, illustrating each group or section with slight notices of structure, arrangement, and general uses, thus anticipating the difficulty of a definition of vegetables, (which seems often too anxiously obtruded on the attention of the beginner,) it is now proposed, in a similar way, to obviate a similar difficulty in the definition of the study, and by an equivalent demonstration of the several departments into which botany has been distributed, to illustrate the science by reference to its subjects; i. e. by taking examples from the several chief groups or sections into which philosophical research, as well as popular consent, has distributed the multitudinous subjects of the vegetable reign, to illustrate the several departments of the science. We then demonstrated existing plants: we now propose to demonstrate the circumstances under which, as plants, they do exist: the one being the subjective, the other the objective view.

Gentlemen: Without reference to obscure archæological researches, the antiquity of our science may fairly be assumed, for plants were the first beings that ever sprang instinct with life on this terraqueous globe, and their culture and their care formed man's earliest employment: since, on the third day of the Creation, so soon as the dry land appeared, when, at the Divine behest, the earth brought forth grass and herbs yielding seed, and the fruit tree yielding fruit, and God saw that these works were good; since the Almighty planted a garden eastward in Eden, and put man, whom he had made, therein to dress it and to keep it; i. e. since out of the ground made the Lord God to grow every tree that is pleasant to the sight and good for food, and, to crown his works, created man to wonder and adore, among the numerous natural miracles which demand his notice and solicit his regard, as there are none that have received, perhaps there are few that have deserved, a greater share of attention than the wonders of the vegetable world, than the trees of the forest and the flowers of the field, which afford the chief and once the only means of sustenance to him and his. Hence some knowledge of such plants as are useful for food, as medicines, or in the arts, must have been almost coeval with our race, at least, congenital with the wants of man; and this knowledge, once empirical, and merely the result of casual observation, was then (as fitted best) called *Herb-craft;* but since that the practice has been reduced to principle, it constitutes a science; it is that branch of natural philosophy and natural history now termed Botany. But, as I shall endeavour to convince you, the botany of the natural philosopher is very different from the botany of the world at large; very different from that specious yet unreal mockery of science, that spurious yet popular and fashionable trifling, which, unconscious of the first principles of vegetable physics, contents itself with superficially scanning the names of plants, esteeming that an end which should never be considered as more than a subordinate, a secondary mean. System is but an instrument, and should never be mistaken for the work it is destined to perform: and such botanists as would confine their studies to mere names and schemes, who burden themselves with tools which are worthless when unused, and which they use not for the purposes for which they were designed, are like scholastic pedants who make language their only study, without reference to the truths which language is destined to reveal; they are always moving, yet never getting on, never getting farther than the threshold of vegetable philosophy; for ever treading as on the wheel, a weary round of never-changing place, of never-ending toil.

Whatever difficulty may have been met with by those who have attempted strictly to define a plant, it might have been reasonably supposed that little could have arisen as to the definition of the science which treats thereof; and that, so long as vegetables formed the subjects of research, so long such researches would be esteemed botanical: but, no; a schism has been mooted even here, which would doubtless excite astonishment, were it a solitary instance of the waywardness of man. On the contrary, however, it is only one of many such, and forms merely an additional proof of what, even without it, daily experience would have sufficed to shew, viz. that no two things more widely differ than do the correct and vulgar acceptations of the terms of science. Hence has arisen the necessity, in the exordium of a discourse, to explain the meanings which should be respectively attached thereto; and thus it is with botany, for this word (Βοτανη), which in the original signifies herb or grass, in modern language is the term applied to the study of plants in general, though some would improperly confine it to the mere art of distinguishing the various particular individuals of the vegetable world. Against this, however, I always have, and ever shall protest; for as well might the meaning be restrained to the original herb or grass as to the diagnosis of the plants alone; for this, which is too often pursued as the sole end and aim, although a part, is but a part, and that not the most important or interesting part of philosophic botany. The systematic branch is no doubt a useful and desirable study, but to those who talk of "Vegetable Physiology and Botany," as if they were sciences distinct, would declare that, with a less absolute abuse of words, might that man be called a botanist who is well acquainted with the structure, functions, and laws of life in vegetables, although he might know not the name of a single plant, than he who could name each plant that grows, if ignorant of vegetable physics, in which we comprehend the anatomy, physiology, chemistry, geography, geology, and other less important branches of the natural history of plants.

Plants are the subjects of botany, their attributes the objects of the science; and, as with other things, these are essential, technical, and accidental; those, universal, general, and special; these, the objects of the study; those, the subjects to be studied; and to the reciprocal elucidation of both, the science, as a whole, is equally devoted. And, first, of the subjects.

The multifarious productions of the vegetable world, which are all the legitimate particulars of botany, have, by common consent, been marshalled in successive ranks, called species, genera, types, and orders; and these have been associated into general classes, and still more general regions, the which, in their aggregate, become the more or less general sections of the universal subject: for example, the vegetable reign, or kingdom, is demarcated into three great regions, comprehending nine large sections, called by different names by different persons, and at different times, but most curiously coincident as to the chief individuals that each rank respectively includes; viz. the Di- Mono- and A-cotyledons of Linnæus and Jussieu; the Exogenæ, Endogenæ, and Cellulares of De Candolle; the Phanero-, Crypto-, and A-gamæ of some modern authors; the Cresses, Leas, and Musts, or Plants, Herbs, and Worts of our provincial dialects; by which names, translatable by the words Cresc-affines, Term-affines, and Myc-affines, we have ventured to propose they should be called.

In consonance with the distribution of plants into three regions and nine classes, subjective botany has already in practice (as I will shew it should be in theory,) been distinguished into various subordinate sciences, on several of which names have been long imposed, although the others are not thus denominated, e. g. Muscologia, or the science of the mosses, which the learned Professor Hooker has made (not his sole, for he has laboured much in every province, but) his more especial study; Algalogia, or the science of the flags or algæ, which Dillwyn, Turner, and the indefatigable Greville, have so industriously investigated; and Mycologia, the science of the fungi, or (as this term should include the whole of the three just-named departments, although, like Muscologia, it be an hybrid word,) we had rather write Fungologia, the science of the mushrooms or fungi, which Fries, and again our own Greville, with Dillenius, Sowerby, Bolton, and others, have reduced to lucid order, and rendered so curiously interesting. The same observations might be made on, and the same principle of subdivision and nomenclature be extended to, the ferns, the grasses, &c.; for the several great sections or classes of plants form sister sciences, analogous to those subdivisions which have been found so useful in zoology, which consists of Ornithology, Herpetology, Ichthyology, Entomology, Helminthology, and so on, each referring especially in turn to the birds, the reptiles, and the fish, insects, worms, &c.; no one zoologist paying equal attention to all, but, having a general knowledge of the whole, dedicates his time especially to one; and much benefit would result to our science, were a similar plan to be adopted by and naturalised amongst us.

Secondly, of the objects of the science, viz. the attributes of the subjects which are their absolute or essential qualities, their comparative relations, and their accidental uses.

The *positive* attributes of plants are those qualities which are *absolute* and *essential* to their physical existence; and the study of these constitutes that section called Organic Botany, or Vegetable Physics.

The *accidental* attributes are those qualities of plants which, although *extrinsic* and not essential to vegetable existence, are often to other beings of *superlative* importance: such are the uses, natural and artificial, and the purposes to which plants either have been, are, or might be applied, as food, as medicines, or in the arts. This section comprehends several subordinate sciences, which in the aggregate form Economic Botany, or the study of Vegetable Utilities.

The *technical* or *comparative* spring from, and are founded upon, the essential and accidental attributes just referred to. This section of the science includes the imposition of names, and the philosophical application of all the various means by which plants are interdistinguished among themselves, and contradistinguished from other coordinate physical existences. This department being chiefly devoted to the theory and practice of arrangement, has hence been termed Systematic Botany, or the study of Vegetable Diagnosis.

The distribution of our subject is therefore trine, and the distribution of our object is threefold also; for, as plants associate naturally into three great regions, so likewise their attributes are

1. Absolute, essential, or positive;
2. Extrinsic, accidental, or superlative;
3. Relative, technical, or comparative.

From these statements it is obvious that botany should be studied both in an objective and in a subjective light, and that each severally initiates a different scheme of investigation. As both courses must be followed, it little matters (as we have already said,) to which precedence may be given; for, although the designs are different, the means are similar and the end the same.

In the subjective scheme, each individual or particular group is uninterruptedly examined in all its aspects, viewed in every position, and submitted to every light; i. e. structure is anatomically examined, functions physiologically investigated, composition chemically considered, &c.: hence, the same subject is contemplated under all its phases before another is submitted to examination; while, on the contrary, in the objective view, a similar examination is uninterruptedly pursued through an entire series of individuals, through the whole vegetable reign, or the whole organic realm: that is, the anatomy of all is investigated, then the physiology, then the chemistry, and so forth: thus giving rise, as before, each to its respective science; here, of vegetable anatomy, physiology, chemistry, and so on; there, of fungology, algalogy, muscology, &c.; in the one case, applicable to the distribution of the subjects; in the other, to that of the objects of vegetable philosophy.

From one of these schemes pursued to its full extent, as referrible to nature as a whole, result the general sciences of botany, zoology, &c., which investigate respectively all the attributes of each kingdom, or of each section separately, both of the organic and of the inorganic world: from the other spring the comparable sciences of general or comparative anatomy, physiology, chemistry, and so forth, which, under the heads of human, comparative, and vegetable anatomy and physiology, of organic and inorganic chemistry, investigate respectively the structure, the functions, the constituents, and all the other attributes of all existing beings. In the one scheme, we view all things in the same light; in the other, the same thing in all lights; or, to take an illustration from my legal colleague, in the one system we examine each witness separately by a succession of counsel, before we call the next; and in the other we bring a succession of witnesses to be examined by the same counsel, before we transfer them to another court, or permit them to be examined by another counsel;—or, to borrow a figure from the chemical professor, in the first we bring a succession of tests to act upon the same substance, and in the other a succession of substances to be tried by the same test.

From this it will appear that botany, as a science, may be described, on the one hand, as subjective or objective, and, on the other, as theoretical, practical, or mixed, and either (according to its extent,) universal, general, or particular: e. g. plants may be studied either in immediate connexion with other natural existences, when the investigation of their structure, their functions, their alliances, their properties, their habits, and their uses, become integral parts of the general sciences of anatomy, physiology, chemistry, geography, geology, and so on, or the several parts of these general sciences which relate to plants, without being rudely severed, may be conveniently distinguished and especially allied; so that, when the anatomy, physiology, chemistry, geography, geology, arrangement, habits, uses, &c. of plants are conjunctively studied, they will become the several objective branches of universal botany, and the various pursuits of the philosophic botanist.

Nor in such pursuits, discursive as the list may sound, does the botanist wander from his rightful path; neither, when encroaching, does he trespass with unbidden foot, nor presume an unwelcome guest upon the provinces or the privileges of his colleagues; for, whatever his science gains, it imparts as much, or more; the advantages of communion are reciprocal, and it is indeed one of the highest and most valued privileges of philosophy, that each branch gains more than it gives by cherishing this system of liberal association. Things which should thus be inseparably conjoined, let none essay then rudely to tear asunder; for, although a distinct consideration of the several departments of natural history and philosophy becomes, from the extent of the study, indispensable, still the division is not the less artificial, and continual reference must be made from each to the facts and truths included in the other.

Now, what is true of nature as a whole, and of the vegetable kingdom as one first grand section, is true of each region and class of plants in general, as well as of each individual species and variety in particular; thus giving rise, according to their relative extent, to general and special botany: for, as universal botany consists of the investigation of all plants, in all circumstances, general botany consists of a similar examination of the general types or classes, and special botany of a like research into any particular group or species. Hence have we those useful and elaborate monographs, and those more succinct yet frequent essays, with which our journals of science and literature teem, which do so much honour to their several authors, and are of such vital importance to the onward progress of philosophy; for these are, as it were, the only sure foundation-stones, thus raised by the miners of science from the quarries of truth, with which the general naturalist or architect can build the temple of human knowledge.

Among the most valuable and beautiful of such special works, I cannot omit the mention of Lambert's "Pinus," one of the most splendid volumes that ever issued from the botanic press, or of the Duke of Bedford's "Salictum Woburnense;" Spix and Martius on the Palms, Michaux on the Oaks of America, Lindley on the Orchides and Roses, and Brown and De Candolle on many sections, with numerous other similar productions which crowd upon the recollection, but of which time will not now permit even a bare catalogue to be made. To the Transactions of the Linnæan, Horticultural, and other learned Societies; to the practical cultivators of indigenous and exotic plants, among whom Knight, Barclay, the Loddidges, the Society of Apothecaries, and others, deserve honourable mention; as well as to the journals of travellers, the special departments of botany likewise stand deeply indebted. Few have ever done more for natural science than Humboldt and Bonpland; Hancock, Douglass, and Wallich claim also grateful notice: the latter more especially from us, as through him the Honourable East India Company have presented to the Herbarium of this College, (the botanical museum of which we are very anxious to render a useful place of reference for students,) a valuable selection from the plants collected by him under their auspices in India; on a scale of magnificence and with a spirit of liberality truly oriental; for the Company, after the plants had been collected, freighted a vessel to bring them home, and have subsequently distributed the duplicates as presents to the learned throughout the whole of Europe. But to return from this digression:

Each of these modes of investigation to which I have referred has advantages peculiarly its own: the one leads to enlarged and liberal views of the general laws which regulate the whole, the other ensures an intimate acquaintance with the habits and characters of particular individuals, and, as always is the case, the more comprehensive the less defined, and the less discursive the more decided, are our views. Hence neither of these methods should be neglected, neither be exclusively pursued, but both be judiciously cherished and rendered helps mete for each other; and in the lectures to be delivered in this College I propose to combine, as far as may be, the advantages of both.

Let me then repeat, that botany is one grand division of natural knowledge, distinguished as a separate science: it consists of three subordinate departments, all of them intimately allied with each other, and closely connected with the correlative general sciences, of which indeed, under another view, they severally form parts. Thus philosophic botany, or the true science of plants, consists not merely either of an account of their structure and their functions, or in a detail of their names, their characters, and their arrangement, or in a history of their habitudes and uses, but comprises all; and hence the three great subdivisions of the science into organic botany, or vegetable physics; systematic botany, or vegetable diagnosis; and economic botany, or vegetable utilities: and these several topics, each in itself important, but too often disconnectedly pursued, lose much by disunion both of interest and value: this should never be forgotten by those who wish to study philosophically the principles, the relations, and the purposes of botany.

Gentlemen, I have now discussed, in general terms, the theory of botany; I have compressed the fruits of much reflection into as short a space as a due regard for perspicuity left possible; and yet I fear, that to such as come fresh and unprepared for any abstract views of a demonstrative science, these truths may have seemed tedious, from their length; although, if obscure, they must have been made so merely from their brevity. I would fain have been more copious in the details, but I fear that, even as it is, I have detained you longer from the demonstrations than is politic in an introductory address. Let us, then, at once begin our practical illustrations; and first of the principles of the science, better known as Organic Botany, or the study of Vegetable Physics; which, being a very extensive province, the maxim "divide and conquer," which induced the first distinction of the science into the subjective and the objective views, and the subsequent division of both into several branches, still persuades to a further distribution of each branch into various subordinate departments: and hence vegetable physics, accordingly as it treats of the several essential attributes of plants, gives rise to the structural, functional, chemical, mechanical, zoological, geographical, and geological sections, each relating especially to the topic its name imports.

And first of Structural Botany, or Vegetable Anatomy, or, as the supremacy of nomenclature would have it, *Phytanatomy.*

Of structural botany, a very extensive and important section, and one to be fully followed out hereafter, time will permit me to offer now but very meagre illustrations: we must content ourselves with almost solitary examples both of external and of internal structure.

As the most notorious of the external attributes of plants, I select their size and figure, for in both the difference is extreme. With respect to size, we have plants on the one hand almost, and others altogether, invisible to the naked eye, and not improbably some that even elude the vigilance of the doublet lens; while, as contrasts to these infinitesimals of vitality, we have palms, and pines, and oaks, from one to two and three, or even, as some report, four hundred feet in height, and of proportionable thickness. "Miracula fugiunt" often forms a comment to the relation of marvels such as these, and true enough it is that wonders, like the visible horizon, do frequently recede as we journey towards them, and often evade our practical researches; but of the above the evidences are within our reach, and in fact are now before us: for this black dust, known familiarly as the smut in wheat, and which is so small in each individual plant that it becomes conspicuous only by their association, and which, small as it is, is not the smallest species; for this is a sample of the Reticularia maxima, and yet each microcosm contains, as Fries (a mycologist of no mean celebrity) affirms, 10,000,000 sporules, i. e. embryo plants, equivalent to seeds, or rather to buds or offsets. So subtle are these minute vegetable existences, that they elude our ordinary vision, and are scarcely seen unless when in multitudes, and then they resemble smoke, and are raised like vapour into the air. Contrast these and many similar protophytes, or even the microscopic yet complicated phasca, so curious in their minuteness, with the royal oak and the lordly pine, this measuring three hundred feet in height, that six-and-twenty yards in girth. Of course, such specimens cannot be stored in our herbaria; yet the Erie walnut, which measured thirty-six feet in circumference at the level of the ground, was seen by many of you in this city only a few months ago, and I believe it is to be seen, by any one who wishes it, at the present time; but, above all, the Cowthorpe oak is still living with us, and probably uniting by its existence three millennia together; for the old oak in Salcey wood, which is little more than half its size, has been computed to have lived upwards of 1500 years.

The form of plants is not less various than their size: some possess roots, and stems, and branches of different structures, bearing multitudes of thorns or prickles, with leaves, tendrils, flowers, and fruit, in every conceivable variety; whilst others, as the Truffle, consist apparently of nothing more than root; others, as the Testudinaria, seem little else than shapeless trunks; and others, as the Aphyteia and Rafflesia, consist of a flower alone: those having neither stem, nor leaf, nor flower; and these having flower only. But this is a subject so entrancing, that I must tear myself at once from the discussion; for I dare not now dilate on the laws which regulate the development and the non-development of these several parts, with their extraordinary transformations into each other; as of the foliage successively into scale, tendril, prickle, leaf-stalk, leaf, flower, and fruit; for the substance of the peach, the pear, the apple, and the cherry is only the disguised and metamorphosed substance of a leaf, as these specimens and diagrams will prove.

A knowledge of the internal structure of the vegetable body assists greatly in explaining the modifications of its external form; but not only this, they reciprocally become indexes to each other, as certain internal structures only can assume certain external configurations; at least, we so conclude from the circumstance of their always being found in combination. But what imports it, methinks I hear some caviller object, that we should know the shape, the disposition, and the contents of the cells and tubes of the vegetable frame? I will answer the supposed objector by an illustration; for one fact, in my mind, will far outweigh a volume of assertion. All wood is tubular and cellular, and the different weight, colour, taste, smell, &c. of oak, ebony, poplar, cedar, sandal, and so forth, depend not on the ligneous structure itself, but on the matter the cells contain; for, if ebony be steeped in any fluid which will dissolve the black matter with which its cells are filled, it will become as light and pale as poplar. But to the example. There are two, if not three, species of British oak, (the third species is by some, however, considered only as a variety,) one of these alone produces strong and lasting timber fit for naval purposes, i. e. which will endure unchanged the transitions from wet to dry, from heat to cold, and remain unhurt between wind and water. This difference depends on the tubes just mentioned conveying to the cells of which the mass of wood consists, a substance differing in solubility in the different species; so that, when the timber of the one is wet, part of the inspissated extract is dissolved and borne away; and when this is repeatedly done, the cells become more and more void, and the timber light and spongy, so that, during cold weather, the water within it freezing and becoming expanded, the cells and tubes are ruptured, and consequently more readily let in fresh water and let out the solid matter it dissolves; and these successive crops of icicles soon form chinks and rents, extending for many feet. Now, oak is frequently contracted for in building ships, and mill-work, floodgates, locks, and so forth, merely as oak, and often, either through ignorance or fraud, the perishable timber is purveyed instead of the enduring wood: but a knowledge of vegetable structure can, by the aid of a very simple experiment, (the manipulations of which I have described in the thirteenth number of the Journal of Science,) easily detect the fallacy or fraud.

From the positive characters discovered by an investigation of the external and internal structure of plants are deduced the comparative or diagnostic signs which enable the systematic botanist to distinguish plants from each other, and give rise to the second department of our science; of which hereafter. But as the subject of the oak has been so far discussed, it may not be amiss to anticipate an illustration, and shew how structural peculiarities will enable the forester to distinguish between the qualities of the timbers before he fells the trees, or rather, in fact, to predict the kind of wood an oak will form, even while the sapling is just springing from the seed: for it is preposterous to contend that plantations should be raised and nurtured through centuries, and then, at the end of two or three hundred years, the fact should be discovered that such oaks are unfit for shipbuilding, and the first notice of this be from the decay of the vessels, even while upon the stocks. I speak not unadvisedly, nor do I put a case of bare possibility; I merely relate a notorious fact. Plantations of the wrong kind of oak have been made in various parts of this essentially oak-growing and ship-building country, and vessels built of such timber as that to which I have alluded *have split and rotted* on the stocks, and have been obliged to undergo a thorough repair, even before they have been launched. What a lamentable tale it is to read or hear, that a vessel of 120 guns, and which must have cost 120,000*l.*, has been condemned and sold for 25*l.*, as last week's journals tell us was the case, and this, as they report, without having ever seen any actual service. Indeed, the rapid decay of many modern-built vessels, and hence much of the heavy expense of our navy, has been, with some shew of reason, attributed to the use of immature and ill-chosen wood, the applicability of which might easily have been tested, had not botanic knowledge been absent from situations where it ought not to have been found wanting.

But of this enough. I must now hasten to give an illustration or two of Functional Botany, generally called Vegetable Physiology, *Phytology*, or *Phytophysiology*.

The functions of plants are either special, as relating to their own well-being, or general, as having an equally extensive importance to other co-ordinate existences. Thus, the absorption of nutritious fluid by the roots, its ascent through the stem to the leaves, its circulation and assimilation to the substance of the vegetable, although perhaps not wholly designed to benefit the individual alone, are chiefly and primarily so: while the transpiration of plants, and their action on atmospheric air, are functions of equal, if not greater importance to animals, than they are to the plants themselves.

The motion of the sap is ocularly demonstrable; and here is a specimen of Chara so placed beneath the microscope, that the currents of sap traversing its various parts are as evident to our senses as the current of a river, or the circulation in the foot of a frog. Here also are diagrams of its course in the stipules of the Ficus elastica, and the water plantain.

The respiration of plants seems to have been generally misunderstood from this function having been confounded with their digestion, both being parts, in fact, of one great process, that of assimilation; although respiration would seem to perform some other important duty, perhaps in maintaining the irritability of the vegetable. This distinction I endeavoured practically to make out, and a summary of an extended course of experiments, undertaken with this view, were published about two years ago, in the first number of the Journal of the Royal Institution, the result of which may be shortly stated thus: that plants always deteriorate atmospheric air by their respiration, but that by their digestion they much more than compensate for this deterioration; and that, on the whole, they tend to preserve the equilibrium which combustion and the respiration of animals have a tendency to disturb.

Of the light which modern chemistry has thrown on some intricate parts of botany, I cannot speak in too grateful terms, however warm my acknowledgments may seem; for by its means we are enabled to explain many physiological phenomena which, without its aid, were utterly inexplicable: e. g. it has been a constant theme of wonder (and remember that it is not the less a miracle now that we can trace back one further link in the chain of events, for physical causes are but effects; it has been, I repeat, a constant theme of wonder) that from the same soil plants should elaborate such different principles; that, fed, sustained, supported, by the same earth, air, and water, one should produce starch, another gum, a third sugar, and so on; that some should abound with bland, others with highly aromatic, essential oils, such as the olive, the almond, cinnamon, cajeput, and so forth. That here we should find the most useful medicines, and there the most deadly poisons; as, for example, bark, opium, and rhubarb, contrasted with prussic acid, the upas tieuté, and woorara.

But not only is it strange that by different plants such different products should be formed from the same materials, or that the same plant, in its different parts, should elaborate many of these varieties; as, for example, a tasteless, harmless oil in the kernel of the bitter almond, and a destructive poison in its outer skin, and so forth, as in the cassava, poppy, and many other similar cases; but it is passing strange that the same parts of the same plant should be at different times so very different in their sensible qualities; at one time consisting almost wholly of insipid lignin, at another being as sour as verjuice, and anon abounding with sugar, impregnated with some delicious aroma.

Phytochymics, discussing the influence of vegetable life on matter, and examining the proximate and ultimate constituents of plants, both while growing and when alive no longer, has thrown much very important light on these obscure and interesting subjects; for hence it is we have learned that, however various the products of the vegetable world may be, (and various to an almost overpowering extent they are,) still all, even the most distinct, consist of charcoal and water, the difference depending upon the ever-varying proportions in which the former is combined with the two elements of the latter: and this is a constitution which can easily be shewn, for I need not advertise my present audience that compound bodies maintain their composite existence by virtue of an attraction which holds their principles or elements together, which is found to be of different degrees of energy between different constituents: so that, if to starch, or gum, or sugar, another substance be added which has a greater attraction either for the charcoal or the water, or for either of the elements of the latter, than they have respectively for each other, the original substance will be decomposed, and its elements enter into new combinations, or be in part or on the whole set free; or, should the added substance have an attraction for only a small quantity of the oxygen, or the hydrogen, or the charcoal, then such part only will be abstracted, and the remainder left in such proportions as to form the other proximate principles alluded to. Hence is it then, that in the growth of plants, in the ripening of fruit, and so forth, that lignin changes into acids of different kinds, acids into sugar, and so on: and thus, indeed, sugar has been made from old rags, tan from sawdust, vinegar from wood, gum from starch, oxalic acid from sugar and from offal, &c. &c.; the three last of which processes are carried on in this country to a great extent, and with much economical advantage.

To Vegetable Mechanics so little attention has hitherto been paid, that I am reluctant to enter on almost untrodden ground, and yet I am unwilling to pass wholly without notice a theme so important and interesting as that which discusses the evidences of design which are so remarkable in the structure of vegetables, and those mechanical advantages which they reciprocally impart and receive in their relative connections. Let one or two examples of these suffice: and I would direct your attention, in particular, to the mechanical advantages which the feeble radicle gains by its position within the valves of its stony prison, the gates of which it can thus unclose with ease, weak as it seems; while, strong as we comparatively are, it often requires no little exertion, as in the peach, the plum, &c., to crush the shell or to rend the sides asunder. The stings of the nettle are also most curiously constructed: each stimulus being a hollow stilet, something like the fang of a rattlesnake, the channel through which communicates with a reservoir, into which a gland at its base pours an acrid fluid, which, when any thing touches the leaf, is compressed, and the fluid, rising through the duct, escapes through an opening at the side of the style near its point, and thus is lodged in the puncture the instrument has made. The Valisneria, the Cyclamen, the Utricularia, and a variety of other plants, exhibit mechanical contrivances equally beautiful, and equally well adapted to fulfil the purposes for which they were evidently designed; but of these more fully in the hereafter lectures.

Botanical Geography, the next point of consideration, is a topic replete with interest; for here we learn how much plants affect and are affected by climate, both physical and geographical, and how they vary according to latitude, longitude, and altitude; for different countries, even in the same or similar parallels, often have vegetations of entirely different characters.

This is a study of such recent date, that the present generation may almost esteem it a science of their own. To the naturalist the truths thus learned are very valuable; and to the medical philosopher it is a study peculiarly important, as the presence or absence of certain plants will often reveal to the eye of science the healthiness or insalubrity of untried or suspected districts; and hence botanical topography not unfrequently becomes one of the surest guides to the local presence or absence of various diseases.

Thus, the colonist should never bivouac nor fix his residence where the Arundo Phragmites flourishes; as it and the Glyceria fluitans and G. aquatica are infallible indications of swampy, marshy districts, and of the probable presence of malaria, even although the tract, as in summer, may seem dry, and be apparently salubrious. A late traveller in Syria thus was warned by the natives not to pitch his tent on the spot that he had selected on account of the luxuriance of the herbage, if he valued his life, or wished to escape a severe attack of fever: this malign influence, however, they seemed erroneously to attribute to the growth of the plants, but of which, in truth, the luxuriant herbage was the index only. Thus also was the indigenous origin of remittents proved in Gibraltar; and thus the absence of the Laurustinus from the gardens of Switzerland shews the severity of the winter there, as the luxuriance of the vintage proves the warmth of the Swiss summer. Again, the barrenness of the apricots and the vines, while the myrtles and the camellias flourish in the open air, will attest the equability of the temperature in Devonshire and Cornwall throughout the year; the heat of the summer being as insufficient to perfect those, as the mildness of the winter is favorable to these: but of examples there would be no end.

Botanical Topography, which treats of the station as well as the habitation of vegetables, includes much knowledge of extreme importance; and even the more special topography of parasitic plants is not wholly destitute of interest or of value; e. g. many of our lichens, fungi, &c. will grow only on certain plants and trees, or often on only especial parts of them, just as many insects inhabit only one genus or species, or only particular parts of the selected habitat: on the oak there are four or five different sorts of galls formed on different parts by different insects, one on the bud, one on the branch, one on the root, one on the leaf-stalk, one on the flower-stalk, one on the leaf itself, &c.; and we are even told that the gall of the upper side of the oak-leaf is the work of a different species of cynips from that which makes the gall upon the under. However that may be, whether the variety is quite so great or not, I do not by experience know; but this I can from observation state, that there are several, probably four or five different galls, apparently produced by different insects. Now, this is not true of the oak only, or of insects alone, the same thing is observable with regard to the parasitic protophytes; and a German botanist has pointed out a very useful application of this knowledge to aid in the discrimination of the true Cinchona from the spurious barks which in commerce are, either from accident or fraud, frequently commingled with it; for he has shewn that one species of lichen is peculiar to and only found on the true officinal cinchona, while the false barks with which it is adulterated, although often covered with other lichens, never bear any of this diagnostic species. Again, I recollect reading that, some years since, in America, a mortal distemper raged with much severity among the people, and was found to be owing to their feeding upon the Zea mays, or Indian corn, as those who did not eat this bread escaped: but why a grain, in general fit for food, should that season have proved so injurious, no one could tell, until a botanist, looking at the subject by the light of science, found that on each grain of corn, just where it had been torn from the ear, a small poisonous fungus grew, to which the fatal influence had all been owing; just as the deleterious effects of cheese are often attributable to a similar plant. But how was the fungus to be prevented from growing? how was the farmer or the miller to avoid the pest, although its source had been detected? They knew not; they were as impotent as before. But the head of him whose eye discovered the bane revealed the antidote; for, as it was found that this fungus only grew on the parts where the grains had been attached to the stalks of the ears, nothing was more easy than to leave the corn unthreshed until it was wanted to be ground into flour. This accordingly was done, and so the plague was stayed; and, in consequence of such a simple application of science to the common purposes of life, large quantities of food were redeemed from destruction, and much human misery providentially averted.

One more illustration, and I have done. The lichens, or aerial algæ, never grow submerged; the fuci, or aquatic algæ, never grow emerged: the same may be said of other plants which are the living demarcations of land and sea; e. g. the samphire (Crithmum maritimum) never grows but on the sea-shore, and yet it never grows within reach of the waves; that is to say, it is never so near as to be covered by the water. It happened not long since that a knowledge of this fact was useful in a way and at a time when botanic knowledge might *a priori* have been expected to be of little practical importance. During a violent storm, in November 1821, a vessel, passing through the English Channel, was driven on shore near Beachey Head, and the whole crew being washed overboard, four escaped from the wreck, only to be delivered, as they thought, to a more lingering and fearful, from its being a more gradual and equally inevitable death; for having, in the darkness of the night, been cast upon the breakers, they found, when they had climbed up these low rocks, that the waves were rapidly encroaching, and they doubted not that, when the tide should be at its height, the whole range would be entirely submerged. The darkness of the night prevented any thing being seen beyond the spot upon which they stood, and which was continually decreasing by the successive encroachments of each successive wave. The violence of the storm left no hope that their feeble voices, even if raised to the uttermost, could be heard on shore; and they knew that, amidst the howling of the blast, they could reach no other ear than that of God. Man could afford them no assistance in such a situation, even if their distress were known. The circle of their existence here seemed gradually lessening before their eyes, their little span of earth gradually contracting to their destruction; already they had receded to the highest points, and already the infuriated waters followed them, flinging over their devoted heads the foremost waves, as heralds of their speedily approaching dissolution. At this moment one of these wretched men,—while they were debating whether they should not in this extremity throw themselves upon the mercy of the waves, hoping to be cast upon some higher ground, as, even if they failed to reach it, a sudden would be better than a lingering death,—in this extremity, one of these despairing creatures, to hold himself more firmly to the rock, grasped a weed, which, even wet as it was, he well knew, as the lightning's sudden flash afforded a momentary glare, was not a fucus, but a root of samphire: samphire is a plant which never grows submerged. This then became more than an olive branch of peace, a messenger of mercy; they knew that He who alone can calm the raging of the seas, at whose voice alone the winds and the waves are still, had placed his landmark, had planted his standard here; and by this sign they were assured that He had said to the wild waste of waters, Hither shalt thou come, and no further. Trusting, then, to the promise of this child of earth, they remained stationary during a dreadful yet then comparatively happy night, and in the morning they were seen from the cliffs above, and conveyed in safety to the shore.

(To be concluded in our next.) Vol. lxviii. p.8.

